# Role of Rb during Neurogenesis and Axonal Guidance in the Developing Olfactory System

**DOI:** 10.3389/fnmol.2016.00081

**Published:** 2016-09-09

**Authors:** Carine Jaafar, Saad Omais, Sawsan Al Lafi, Nadim El Jamal, Mohammad Noubani, Larissa Skaf, Noël Ghanem

**Affiliations:** Department of Biology, American University of BeirutBeirut, Lebanon

**Keywords:** Rb, neurogenesis, olfactory epithelium, olfactory bulb, axonal guidance, signaling molecules, cell survival

## Abstract

The Retinoblastoma protein, Rb, was shown to regulate distinct aspects of neurogenesis in the embryonic and adult brain besides its primary role in cell cycle control. It is still unknown, however, whether Rb is required for tissue morphogenesis and the establishment of synaptic connections between adjacent tissues during development. We have investigated here the role of Rb during development of the olfactory system (OS), which heavily relies on reciprocal interactions between the olfactory epithelium (OE) and the olfactory bulb (OB). We show that mice carrying a telencephalic-specific deletion of Rb display several neurogenic defects in the OS during late development. In the OE, loss of Rb leads to ectopic proliferation of late-born progenitors (Tuj-1+), abnormal radial migration and terminal maturation of olfactory sensory neurons (OSNs). In the OB, deletion of Rb causes severe lamination defects with loss of clear boundaries between distinct layers. Importantly, starting around E15.5 when OB glomerulogenesis is initiated, many OSNs axons that project along the olfactory nerve layer (ONL) fail to properly innervate the nascent bulb, thus resulting in partial loss of connectivity between OE-OB and gradual neuronal degeneration in both tissues peaking at birth. This deficiency correlates with deregulated expressions of two key chemo-repellant molecules, Robo2/Slit1 and Nrp2/Sema3F that control the formation of dorsal-ventral topographic map of OSNs connections with OB glomeruli. This study highlights a critical requirement for Rb during neurogenesis and the establishment of proper synaptic connections inside the OS during development.

## Introduction

The mammalian olfactory system (OS) is a pseudostratified neuroepithelium comprised of the olfactory epithelium (OE) and the olfactory bulb (OB). The OE is made of three cell types: the basal cells (BC; self-renewing cells and transit-amplifying cells) including the horizontal (HBC) and globose (GBC) cells located in the inner basal layer, which is adjacent to the basal lamina, the immature olfactory sensory neurons (iOSNs) found in the intermediate layer that progressively migrate to the apical layer as they become mature (mOSNs), and the supportive sustentacular cells (SUSs) primarily residing in the apical layer (Graziadei and Graziadei, 1979; Nicolay et al., 2006).

The OE lineage is controlled by intrinsic transcription factors that regulate OSN production from basal stem cells and distinct precursors. These include Sox2, a stem/early precursor marker, Mash1, a marker of committed neural precursors, Ngn1, which labels intermediate neural precursors, and NeuroD1-positive neural precursors that give rise to iOSNs expressing β-III tubulin or Tuj-1, which finally mature into mOSNs expressing the olfactory marker protein (OMP) (reviewed in Kam et al., 2014).

Starting around E11.5 until E15, OSNs axons along with a population of “pioneer” neurons together called the “migratory mass” project to the rostral tip of telencephalon and form the presumptive olfactory nerve layer (ONL) that undergoes continuous growth (Valverde et al., 1992; Whitlock and Westerfield, 1998; Raper and Mason, 2010). Around E15, when glomerulogenesis begins in the OB, OSNs axons grow deeper and form synapses with the primary dendrites of the main projection neurons in the OB, the mitral cells (MCs), and the tufted cells (TCs) (Treloar et al., 1999, 2010). These excitatory neurons relay olfactory information by directly innervating olfactory cortical regions (Wilson et al., 2006). Moreover, their excitatory activity is modulated by GABAergic inhibitory neurons, which help synchronize and refine the spatio-temporal tuning of sensory responses (Breton-Provencher and Saghatelyan, 2012).

In the developing mouse OS, odors are detected by distinct subtypes of OSNs, each of which expresses a single type of olfactory receptors (ORs). There is a tight correlation between the position an OSN occupies in the OE and its target glomerulus inside the OB, thus creating a well-defined topographical organization. The formation of such an olfactory map relies on two types of interactions: axon-axon and axon-target interactions, which are controlled by a combination of secreted and adhesive factors including chemo-attractant and chemo-repulsive cues. For instance, the latter type of interactions is mediated by chemical cues derived from target cells and guide OSN axons to reach their destinations. The temporal sequence of OSNs projections specifies the dorsal-ventral topographic order (D/V) in the OB: hence, OSNs positioned in the dorso-medial domain of the OE innervate the dorsal part of the OB and mature early in development while OSNs located in the ventro-lateral domain of the OE project ventrally to the OB and mature later (Astic et al., 1987; Miyamichi et al., 2005). This D/V patterning is primarily controlled by the graded expression of Robo2 and Nrp2, and their specific interactions with their repulsive ligands, Slit1, and Sema3F, respectively (Doetsch, 2003; Cho et al., 2007, 2011; Nguyen-Ba-Charvet et al., 2008; Takahashi et al., 2010; Takeuchi et al., 2010).

The Retinoblastoma gene, Rb, is a tumor suppressor gene that primarily controls entry into cell cycle at the G1-S phase checkpoint by directly interacting and inhibiting the function of transcription factors required for DNA synthesis mainly the E2F family of transcription factors (reviewed in Sage, 2012; Cheffer et al., 2013). Besides this primary role, recent studies have implicated the Rb/E2F pathway in the control of many aspects of neurogenesis in the brain including neuronal differentiation in the ventral cortex (Ghanem et al., 2012), migration in the dorsal and the ventral cortices (Ferguson et al., 2005; McClellan et al., 2007; Ruzhynsky et al., 2007; Ghanem et al., 2012) and survival in the adult olfactory bulb (Naser et al., 2016). Hence, we have shown that the Rb/E2F pathway coordinates the transition from proliferation to differentiation in the ventral cortex through direct modulation of *Dlx1/2* gene expression, and is required for proper maturation and survival of GABAergic and dopaminergic neurons in the developing olfactory bulb (Ghanem et al., 2012). Moreover, Rb was shown to regulate neuronal migration in a cell-autonomous manner through its specific interaction with E2F3 and direct regulation of the guidance molecule, neogenin, in the developing cortex (Ferguson et al., 2005; McClellan and Slack, 2006; McClellan et al., 2007; Andrusiak et al., 2011). It is still unknown, however, whether Rb plays a role during neurogenesis in the OE and in the establishment of synaptic connections between the OB and OE during development. In this study, we tackle these questions and show that Rb controls the number of OSNs produced in the developing OE, and is needed for the formation of proper axo-dendritic connections between OE-OB. This latter defect correlates with the deregulated expressions of key chemo-repellant molecules that control the formation of the D/V topographic map inside the OS.

## Materials and methods

### Mice and tissue preparation

Foxg1-Cre male mice (Hebert and McConnell, 2000) were mated with Rb^floxed/floxed^ females that are maintained on a mixed C57Bl6/FVBN background. The Rb floxed allele has exon 19 flanked by two LoxP sites (Cre-specific sites) (Marino et al., 2000). All animal procedures were carried according to protocols approved by the Institutional Animal Care and Use Committee (IACUC) at the American University of Beirut. Mice were euthanized with Xylasine/Ketamin solution (1.5 μl/g ketamine and 0.25 μl/g Xylasine) and sacrificed by cervical dislocation. Embryonic pups were collected in 1x PBS and their heads fixed in 4% paraformaldehyde (PFA) at 4°C for 4–16h depending on their age. Samples were then washed several times in 1x PBS and cryoprotected using a sucrose gradient in 1x PBS as follows: 12 sucrose (1 day), 18 (1 day), and 22% (3 days). Finally, heads were embedded in OCT medium (Tissue-Tek, Surgipath) and snap frozen in cold isopentane (Sigma Aldrich) on dry ice. Tissue sectioning was performed using a cryostat and 10 μm-thick sections were mounted onto superfrost slides (Thermo Scientific) and stored at −80°C.

### Genotyping

Mice were weaned 20–25 days after birth. Ear and tail pieces were taken from adult mice and embryos for genotyping, respectively. DNA extraction was performed using phenol-chloroform-isoamyl alcohol mixture (Sigma) as per protocol. Screening was performed by PCR using the following primers: *Rb flox primers*: forward 5′ GGCGTGTGCCATCAATG 3′ and reverse 5′ AACTCAAGGGAGACCTG 3′, *Cre primers:* forward 5′ TGACCAGAGTCATCCTTAGCG 3′ and reverse 5′AATGCTTCTGTCCGTTTGCC3′. Rb heterozygous embryos (Bf-1 Cre+/−;Rb flox/+) showed a similar phenotype to wild-type embryos (Bf-1 Cre +/−;Rb+/+) embryos and thus used as controls compared with Rb mutant mice (Bf-1 Cre+/−;Rb flox/flox).

### Bromodeoxyuridine (BrdU) injection

Pregnant females were given a single intraperitoneal BrdU injection 2 h prior to sacrifice at distinct embryonic time-points. BrdU was administered according to body weight (50 mg/kg): 10 mg/ml BrdU solution (Sigma) was prepared using 0.9% NaCl and 0.007N NaOH. For BrdU staining, tissue sections were treated as follows: 45 s incubation in acetone for dehydration followed by 10 min wash in 1 × PBS and incubation in 1N HCl for 20 min at 37°C to denature the DNA. Sections were then neutralized in 0.1M sodium borate (pH = 8.5) (Fisher scientific) for 10 min and washed in 1 × PBS. Slides were processed for immunohistological analyses as described below.

### Cresyl violet staining

Cresyl violet staining procedures were performed as previously described (Sirkin, 1983).

### Immunohistochemistry

Frozen slides were air-dried for 30 min at least and sections blocked for 1–2 h in blocking solution (1% BSA, 0.3% Triton X, 5% normal donkey serum, and 0.1M PBS). Slides were then incubated with primary antibody (ies) that are diluted in blocking solution overnight (O/N) at room temperature (RT). The next day, sections were washed in 1 × PBS 3 times for 10 min each, and incubated with fluorescent secondary antibodies for 2 h at RT. Finally, slides were washed in 1 × PBS for 3 × 5 min, counterstained with Hoechst and mounted in (3:1) PBS/Glycerol solution. For staining nuclear proteins e.g., Sox2, antigen retrieval was performed by treating tissue sections with 10 mM sodium citrate (pH = 6) (Fisher scientific) for 20 min at 95°C prior to incubation with primary antibody(ies).

The following primary antibodies were used: mouse anti-BrdU (1:50; BD Pharmingen), rat anti-BrdU (1:150; Accurate), mouse anti-PSA-NCAM (1:1000; Chemicon), rabbit anti-OMP (1:300; Abcam), rabbit anti-Tuj1 (1:5000; Covance), mouse anti-Bax (6A7) (1:300; Abcam), mouse anti-AC3 (1:500; Cell Signaling), goat anti-Sox2 (1:150, Santa Cruz), rabbit anti-Ki67 (1:500, Cell Marque), goat anti-NeuroD1 (1:100, Santa Cruz), Rabbit anti-PH3 (1:250, Millipore), mouse anti-Reelin (1:750, Calbiochem) and rabbit anti-GABA (1:2000, Sigma). The secondary antibodies (Molecular Probes, Invitrogen) used were: donkey anti-mouse 596 Alexa Fluor (red), donkey anti-rabbit 596 Alexa Fluor (red), goat anti-rat 596 Alexa Fluor (red), donkey anti-rat 596 Alexa Fluor (red), donkey anti-rabbit 488 Alex Fluor (green), donkey anti-goat 596 Alexa Fluor (red) and donkey anti-goat Alexa Fluor 488 (green).

### *In situ* hybridization

*In situ* hybridization was performed as described in Wallace and Raff (1999). Sense and anti-sense riboprobes were synthesized using digoxigenin-labeled UTP (DIG-UTP). Hybridization was performed overnight at 65°C. Signal detection was done using an AP-conjugated anti-DIG antibody (1:2000; Roche) followed by incubation in NBT/BCIP solution (Amresco) at RT. Riboprobes were prepared from cDNA plasmids for *Slit1, Slit2, Robo1*, and *Robo2* (obtained from Dr. Marc Tessier-Lavigne), and Sema3F (obtained from Dr. Joost Verhaagen). 854 bp of the coding sequence of *Nrp2* was amplified by PCR from C57BL/6 E15.5 cDNA library using the following primers: Nrp2, forward primer with T3 promoter sequence 5′ AATTAACCCTCACTAAAGGGGGTGAAGAATGGCTTCAGGTAG 3′ and reverse primer with T7 promoter sequence 5′ TAATACGACTCACTATAGGGATACTCCATGTCATAGCTGGGC 3′. The resulting PCR fragment was purified and used for riboprobe synthesis.

### Western blot

Olfactory epithelia were dissected from E17.5 embryos and protein lysates prepared in Universal Lysis Buffer. Electrophoresis was performed using 8% Tris-Chloride gels and proteins were transferred to polyvinylidene difluoride membranes (Bio-Rad Laboratories, Hercules, CA), and detected using antibodies to total RB (BD-Pharmingen, San Diego, CA, 1:250) and GAPDH (Santa Cruz, 1:1000). The secondary antibodies used are: goat anti-mouse HRP (Santa Cruz 1:2500) and goat anti-rabbit HRP (Santa Cruz, 1:2500).

### Image analyses and cell counts

Fluorescent and bright-field images were captured using an upright Leica microscope (DM6B) with UV light and digital camera. The OB and OE surface areas were measured every 4th sagittal section taken at lateral and medial levels using the Image J software. All cell counts were performed on matching sections along the OS and normalized to the total surface area in mm^2^ as follows: single positive cells and double positive cells were counted every 3rd section at lateral and/or medial levels to cover 100–120 μm thickness in the OE (total of 3–4 sections). Image overlapping and analyses were carried using the Leica Application Suite (LAS X) software and Adobe Photoshop. For statistical analyses, 2 way-ANOVA (ł) and independent samples *t*-tests were applied using the SPSS program with a minimum 95% confidence threshold. All data is presented as the arithmetic mean, plus or minus the standard deviation of the mean (mean ± SD). All results were generated in at least *n* = 3 per genotype.

## Results

### Abnormal development of the OS in Rb conditional knock-out mice

**Figure 1:**
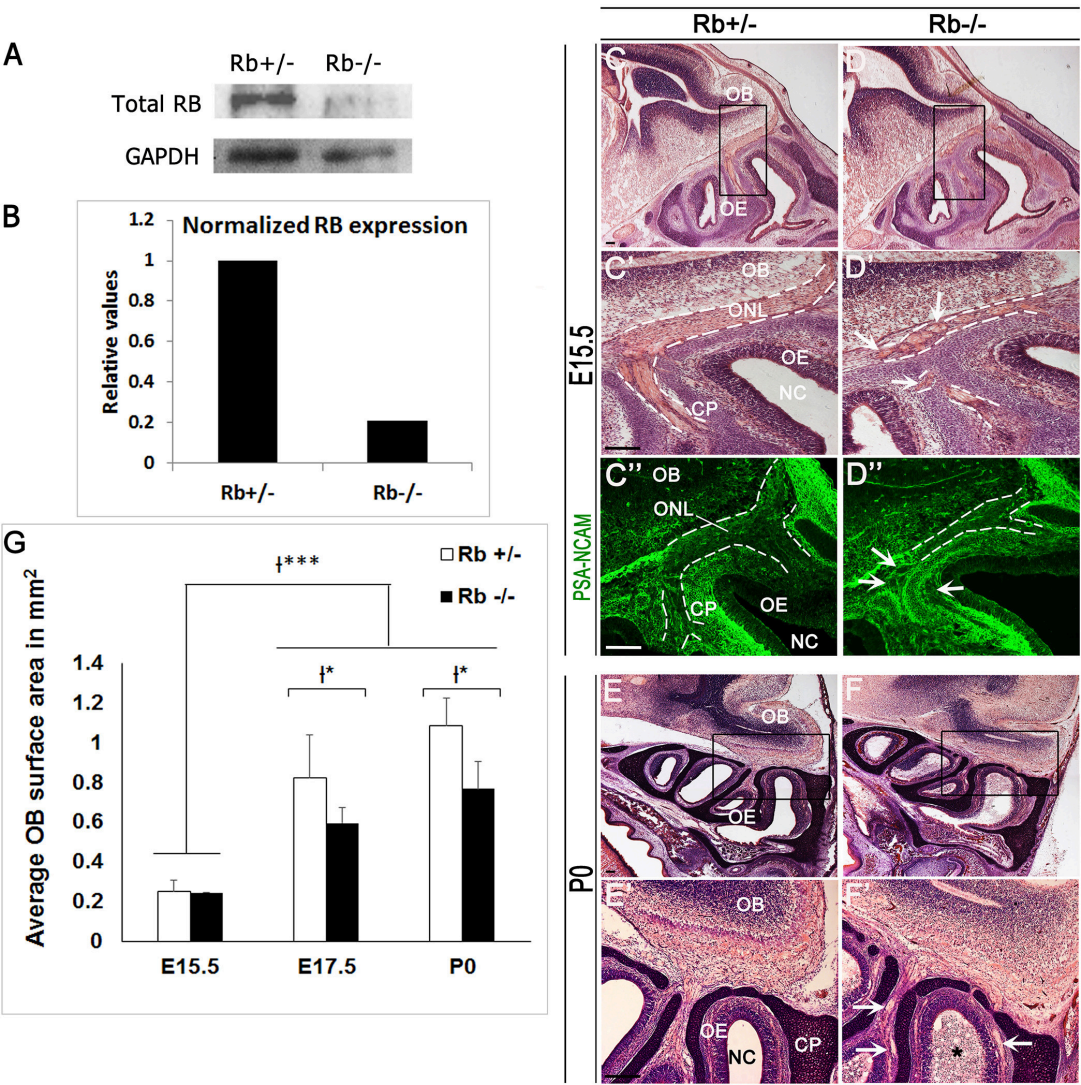
**Abnormal development of the OS in Rb−/− mice. (A)** Western blot analysis performed with protein lysates extracted from olfactory epithelia at E17.5 and showing a successful Rb deletion, and **(B)** 5-fold decrease in normalized Rb expression compared with GAPDH as internal control. **(C–D′,E–F′)** Cresyl-eosin staining performed on sagittal OE sections at E15.5 **(C-D′)** and P0 **(E–F′)**; **(C′,D′,E′,F′)** are higher magnification images of the regions shown in black boxes in **(C–F)**, respectively. **(C**″**–D**″**)** Staining with PSA-NCAM (polysialylated neural cell adhesion molecule), a marker of developing and migrating neurons, illustrating the axonal guidance defect in the ONL in the absence of Rb. Dashed lines in **(C′–D**″**)** delineate the borders of the ONL. White arrows in **(D′,D**″**,F′)** point to misguided OSNs axons that form aberrant round projections inside the ONL at the base of the telencephalon. The asterisk in **(F′)** depicts the presence of detached cells inside the nasal cavity in Rb−/− mice at P0. **(G)** Quantification showing a significant decrease in the OB surface area at E17.5 and P0 in Rb−/− vs. Rb+/− embryos. Error bars represent SD of measurements from *n* = 3 per genotype and asterisks indicate statistical significant difference between genotypes and time-points using 2-way ANOVA tests, ^*^p < 0.05 and ^***^p < 0.001. Scale Bar = 100 μm. OB; Olfactory Bulb, OE; Olfactory Epithelium, NC; Nasal Cavity, ONL; Olfactory Nerve Layer, CP; Cribriform Plate.

**Figure 2:**
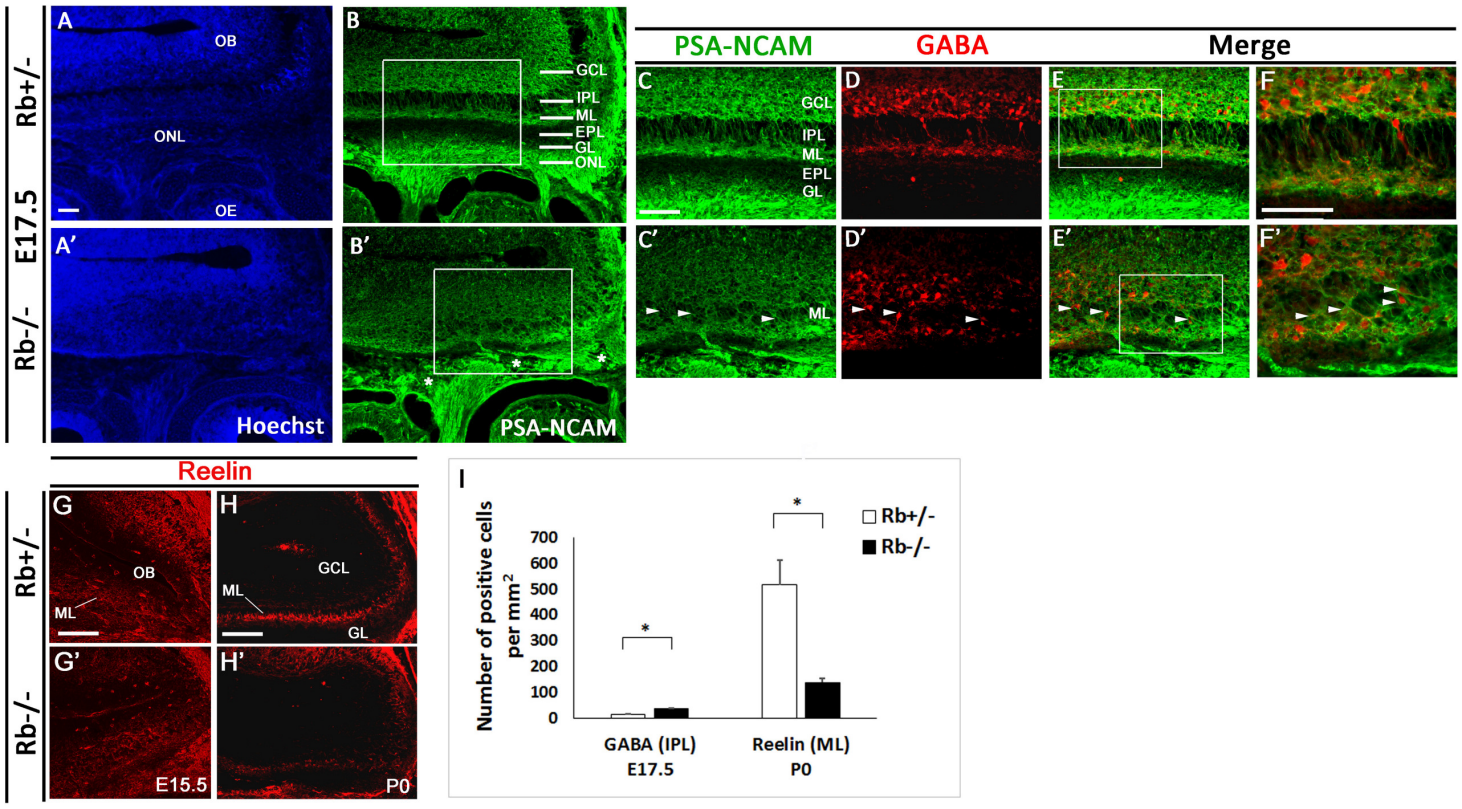
**Lamination defects and loss of mitral cells in the OB in Rb−/− mice during late development**. Immunostaining against PSA-NCAM performed on sagittal sections **(A–B′)** at E17.5 and showing aberrant lamination inside the OB, primarily affecting the IPL, ML, and EPL in the absence of Rb. **(C–E′)** Double staining against PSA-NCAM and GABA (gamma-amino-butyric acid) showing scattered GABAergic interneurons inside the IPL and ML with loss of clear boundaries between these layers in Rb−/− vs. Rb+/− mice (arrowheads in **C′–F**′). ^*^ in **B**′ depicts the presence of axonal guidance defects inside the ONL. **(F,F′)** are higher magnification images of regions shown in **(E,E')**, respectively. **(G–H′)** Staining with Reelin, marker of the proximal dendrites of mitral cells, performed on sagittal sections at E15.5 **(G,G′)** and P0 **(H,H′)** and showing gradual degeneration of the ML in Rb−/− mice compared with Rb+/− mice at birth. **(I)** Graph depicting the numbers of GABAergic neurons mislocalized in the IPL and of Reelin-positive cells in the ML in both genotypes *t*-tests, ^*^p < 0.05. Scale Bar = 100 μm. GCL; Granule Cell Layer, GL; Glomerular Layer, ML; Mitral Layer, IPL; Internal Plexiform Layer, EPL; External Plexiform Layer, ONL; Olfactory Nerve Layer.

We and others have shown that, besides cell cycle control, the Rb/E2F pathway regulates distinct aspects of neurogenesis in the brain such as neuronal differentiation (Ruzhynsky et al., 2007; Ghanem et al., 2012), migration (Ferguson et al., 2005; McClellan et al., 2007; Andrusiak et al., 2011; Ghanem et al., 2012) and survival (Naser et al., 2016). We have investigated here the role for Rb during development of the OS and thus, have generated mice carrying a telencephalic-specific Rb deletion (Rb−/−) by crossing Foxg1-Cre mice (Hebert and McConnell, 2000) and Rb^flox/flox^ mice (Marino et al., 2000). Foxg1, also known as brain factor-1 (BF-1), is a member of the Forkhead transcription factor family and specifically expressed in neural progenitors in the telencephalon and olfactory pit starting around E8.5–E9.5 until birth (Hebert and McConnell, 2000). Rb mutant embryos and Rb heterozygous controls will be thereafter referred to as Rb−/− and Rb+/−, respectively. We first verified that Rb deletion was successful by performing western blot analysis using protein lysates derived from dissected olfactory epithelia at E17.5. Quantification showed a 5-fold decrease in Rb protein expression in Rb−/− embryos compared with controls (Figures 1A,B). Morphological analysis of the developing OS revealed no major abnormalities in the OE and the OB during mid-gestation (E12.5) in the absence of Rb (Supplementary Figure 1). Hence, both tissues undergo simultaneously normal, yet independent, developmental programs during this period as previously described (López-Mascaraque and de Castro, 2002). However, starting around E14.5-E15, when glomerulogenesis begins in the OB, many OSNs axons projecting along the ONL fail to properly connect with the OB in Rb−/− mice compared with control littermates as indicated by cresyl-eosin staining. Thus, they de-fasciculate and create circle-like structures that project aberrantly near the base of the telencephalon (Figures 1C–D′; white arrows in Figure 1D′). Labeling for the polysialylated neural cell adhesion molecule (PSA-NCAM), a marker of developing and migrating neurons, confirmed the presence of severe axonal guidance defects with thinning of the ONL (Figures 1C′′–D′′; dashed lines, and white arrows in Figure 1D′′, and Figure 2B′; asterisks). These defects are exacerbated at birth leading to a disorganized ONL with partial loss of connectivity between OE and OB as well as gradual degeneration of the former tissue as manifested by the presence of many detached and dispersed neuroepithelial cells inside the nasal cavity (Figures 1E–F′; white arrows and black asterisk in Figure 1F′). These results indicate that Rb is needed to establish proper sensory connectivity between OE-OB during late development.

Owing to the axonal pathfinding defects described above, we next examined whether loss of Rb affects OB development. Compared with controls, Rb−/− embryos showed a significant reduction in the size of the OBs between E15 and birth (Figure 1, compare Figures 1C–D and Figures 1E–F, and Figure 1G). Co-staining for PSA-NCAM and γ-aminobutyric acid (GABA), a marker of inhibitory neurons revealed the existence of major lamination defects inside the OB in Rb−/− embryos compared with Rb+/− controls. These are manifested by lack of clear boundaries between the internal plexiform layer (IPL), the mitral cell layer (ML) and the external plexiform layer (EPL) in the absence of Rb at E17.5 (Figures 2A–C′; arrowheads in 2C′). Moreover, many GABAergic neurons that normally reside in the granule cell layer (GCL) as seen in Rb+/− brains are found aberrantly scattered between the presumptive IPL and ML in Rb−/− brains at this age (Figures 2C–F′; arrowheads in Figures 2C′–F′, and Figure 2I). We then examined the expression of Reelin, which labels the proximal dendrites of mitral cells and found that, in Rb−/− embryos, the ML is intact at E15.5 but gradually disintegrates between E17.5 and birth compared to controls (Figures 2G–H′ and Figure 2I). This is likely a secondary defect due to the failure of these projection neurons to establish proper synaptic connections with OSNs axons at earlier stages as reported above. Altogether, our results indicate that Rb is required for laminar patterning and neuronal survival inside the OB, however, these defects may be directly and/or indirectly associated with its loss during OE development.

### Ectopic neuroblast proliferation and decreased survival of newborn OSNs in Rb−/− mice during late development

**Figure 3:**
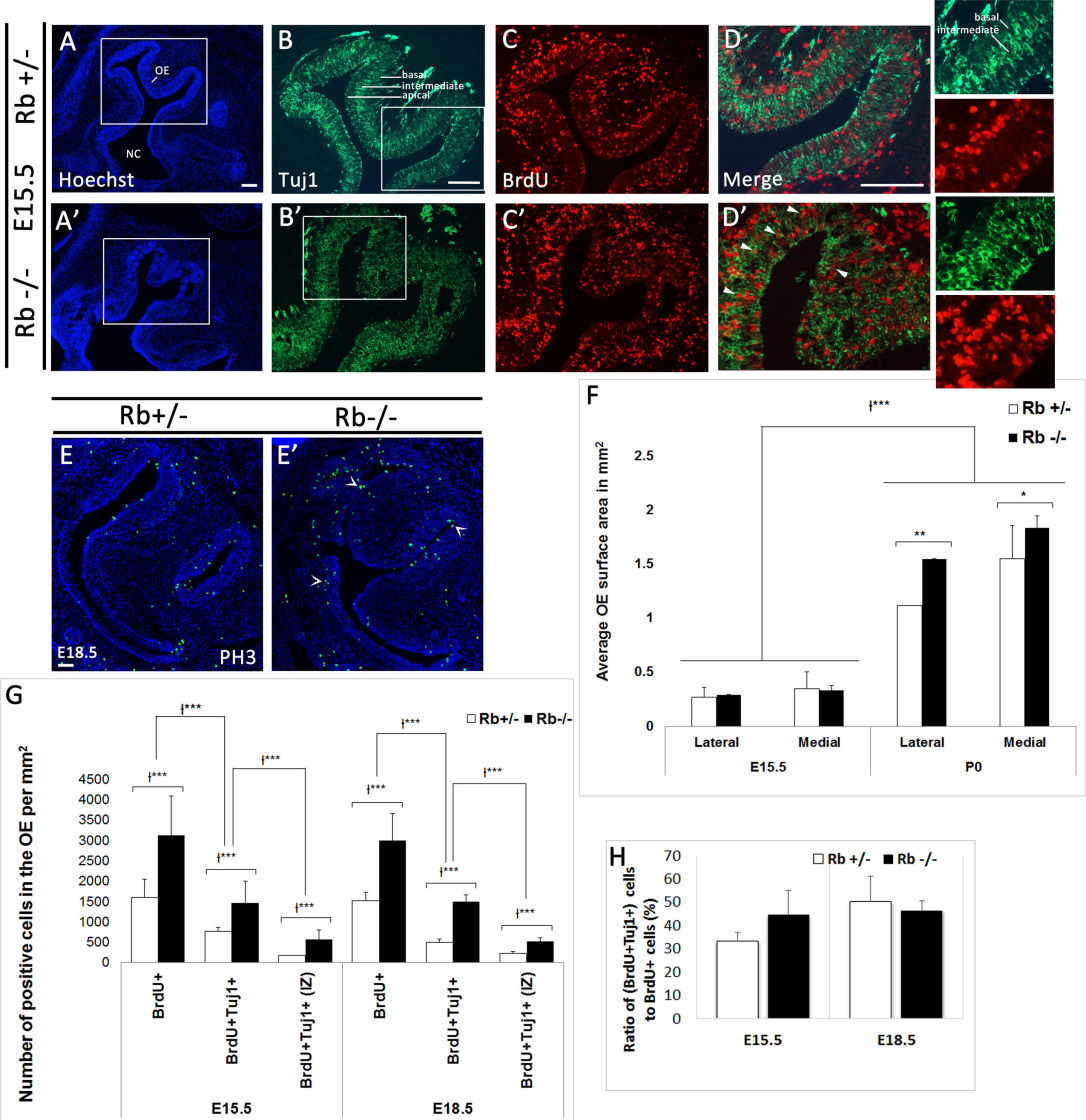
**Rb deletion causes ectopic proliferation and abnormal radial migration of neuroblasts in the developing OE. (A–D′)** Double immunostaining with Hoechst (blue), anti-Tuj1 (green), and anti-BrdU (red) performed on sagittal sections in Rb+/− **(A–D)** vs. Rb−/− **(A′–D′)** embryos at E15.5. **(D,D′)** are higher magnification images of the regions shown in white boxes in **(B,B′)**, respectively. Loss of Rb leads to increased progenitor proliferation and abnormal radial migration in the OE. Many (Brdu+Tuj1+) cells are found scattered in the intermediate zone (IZ) in Rb−/− OE (arrowheads in **D**′) with randomly orientated neurites (**D**′; green and red insets) compared with controls where they are primarily located in the apical and basal layers with parallel neurite orientation (insets in **D**). **(E,E′)** Immunostaining with PH3 (phospho-histone H3, M phase marker) performed at E18.5 and showing increase in the number of post-mitotic cells in Rb−/− vs. Rb+/− embryos (arrowheads in **E**′), thus suggesting the absence of major defects in cell cycle exit. **(F)** Quantification revealed a significant increase in the average surface area of OE in Rb−/− embryos vs. Rb+/− control littermates at P0, consistent with the increased proliferation described above. **(G)** Quantifications of BrdU+, BrdU+Tuj1+ and BrdU+Tuj1+ (IZ, intermediate zone) cells in the OE at E15.5 and E18.5 showing 2–3-fold increase in the numbers of these cells in the absence of Rb (see text for detail). **(H)** Graph showing no change in the ratios of double positive cells to BrdU+ cells between genotypes at the ages examined **(H)**. Error bars represent SD of measurements from *n* = 3 per genotype and asterisks indicate a statistical significant difference between genotypes using 2-way ANOVA tests, ^*^p < 0.05, ^**^p < 0.01, ^***^p < 0.001. Scale bars = 100 μm.

Given the critical role played by Rb at distinct stages of neurogenesis in the telencephalon, we examined whether Rb also regulates neurogenesis in the developing OE. First, we assessed progenitor proliferation following a 2 h-BrdU pulse before sacrifice at distinct embryonic ages. Compared with Rb+/− embryos, Rb−/− embryos showed 1.95- and 1.97-fold increase in the number of BrdU-positive cells detected inside the OE at E15.5 and E18.5, respectively (Figures 3A,A′,C,C′,G, and Supplementary Figure 2; BrdU+: Rb+/− vs. Rb−/−, 1601+/−446 vs. 3129+/−968, E15.5, and 1520+/−202 vs. 3005+/−658, E18.5, *p* = 0.00005). Moreover, there was a proportional increase in the number of BrdU+ cells co-expressing the early differentiation marker Tuj1 (β-III tubulin) with no change in the ratio of (BrdU+Tuj1+) cells over the number of BrdU+ cells between genotypes [(Figures 3B–D′,G,H and Supplementary Figure 2; (BrdU+Tuj1+): Rb+/− vs. Rb−/−, 773+/−100 vs. 1474+/−527, 1.9-fold increase at E15.5, and 505+/−76 vs. 1498+/−162, 2.96-fold increase at E18.5, *p* = 0.00005)]. Of note, many (BrdU+Tuj1+) cells were found ectopically proliferating in the intermediate zone (IZ) [(Figures 3D,D′; arrowheads and red inset in Figure 3D′, and Figure 3G; (BrdU+Tuj1+) in IZ: Rb+/− vs. Rb−/−, 174+/−11 vs. 573+/−236, 3.29-fold increase at E15.5, and 221+/−58 vs. 519+/−85, 2.34-fold increase at E18.5, *p* = 0.00005)]. Moreover, unlike in Rb+/− embryos where all Tuj1+ neuroblasts project their extensions in parallel and well-aligned orientation between the apical and basal layers of the epithelium, many Tuj1+ cells were disoriented and randomly dispersed between these layers in the Rb−/− OE, suggesting the presence of a radial migration defect (Figures 3D,D′; green insets). Despite the ectopic neuroblast proliferation, loss of Rb did not seem to result in major cell cycle exit defect since the number of PH3-positive cells was also found to be increased in Rb−/− vs. Rb+/− OE at E18.5 (Figures 3E,E′; arrowheads in Figure 3E′: PH3+: Rb+/− vs. Rb−/−, 602+/−157 vs. 801+/−60, *p* = 0.01, 1.33-fold increase). However, this does not rule out that some progenitor cells failed to exit the cell cycle and underwent apoptosis at earlier time-points. In addition, the average surface area of the OE along the latero-medial axis was significantly expanded around birth in the absence of Rb (Figure 3F: OE surface area in mm^2^ at P0; Rb+/− vs. Rb−/−, 1.12+/−0.078 vs. 1.55+/−0.1, *p* = 0.0051, 1.38-fold increase at lateral level, and 1.45+/−0.17 vs. 1.84+/−0.3, *p* = 0.021, 1.27-fold increase at medial level).

**Figure 4:**
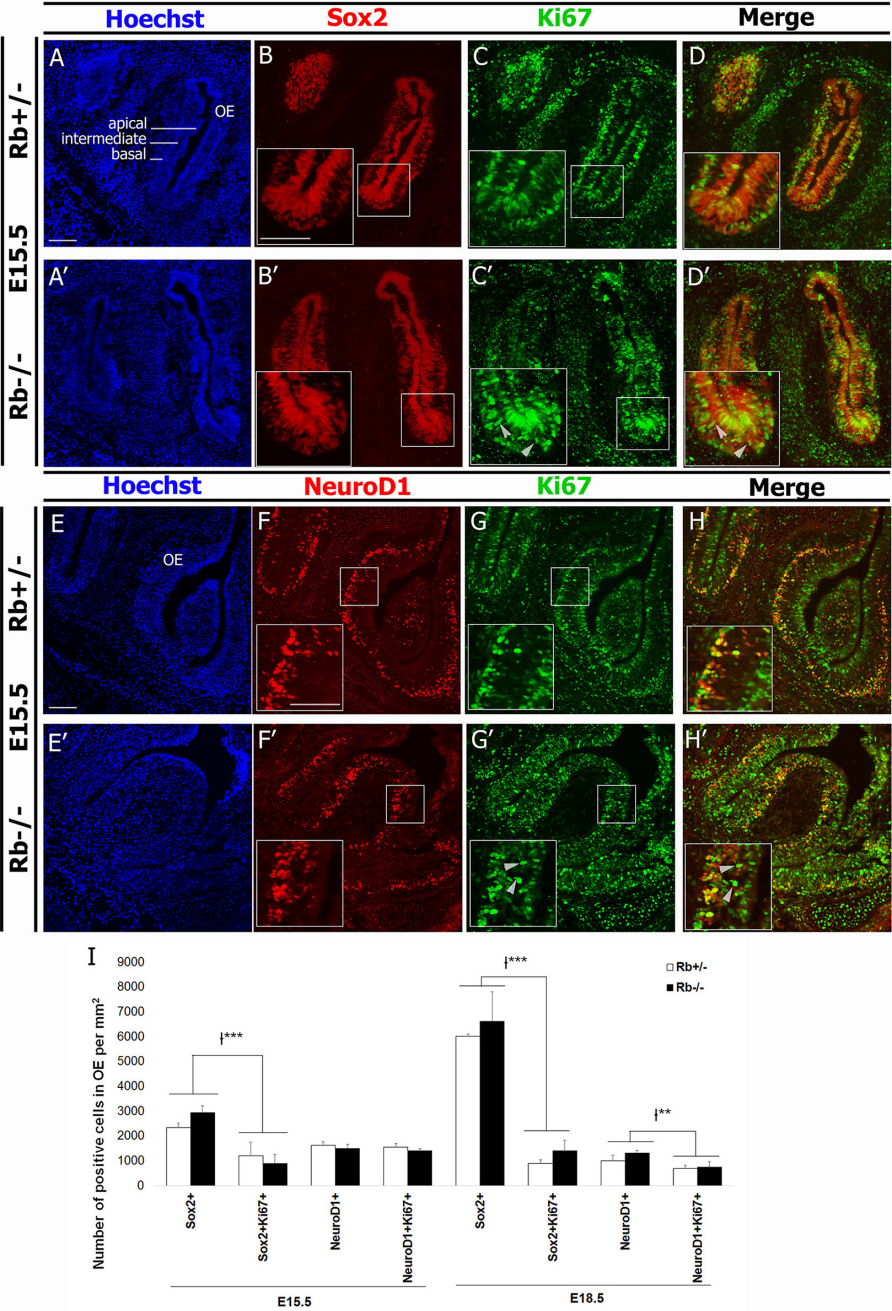
**Loss of Rb does not affect proliferation of stem cells and early precursors in the OE**. **(A–D′)** Double immunostaining with anti-Sox2 (marker of stem cells/early precursors) and anti-Ki67 (proliferation marker), and **(E–H′)** anti-NeuroD1 (marker for intermediate neural precursors) and Ki67 performed on sagittal sections in the OE at E15.5. In the absence of Rb, there is ectopic proliferation of Ki67+ cells inside the IZ as previously shown with BrdU+ cells in the same region (**C′,G**′; arrowheads). However, the numbers of proliferating Sox2+ and NeuroD1+ cells are not affected by loss of Rb. **(I)** Quantification of this data was performed at medial level in the OE at E15.5 and E18.5. Error bars represent SD of measurements from *n* = 3 per genotype and asterisks indicate a statistical significant difference between genotypes using 2-way ANOVA tests, ^**^p < 0.01, ^***^p < 0.001. Scale bars = 100 μm.

In order to assess whether Rb controls proliferation at earlier stages of the OE lineage, e.g., stem cells and/or precursor cells, we examined the expressions of Sox2, a stem cell/early progenitor marker, and NeuroD1, a marker of intermediate neural precursors along with the expression of Ki67 to assess cell proliferation. Consistent with our BrdU data, we detected a significant increase in the number of Ki67+ cells that are ectopically proliferating in the IZ (Figures 4C,C′,G,G′; arrowheads in insets). However, we found no significant difference in the numbers of Sox2+, NeuroD1+, (Sox2+Ki67+), and (NeuroD1+Ki67+) cells inside the OE between genotypes at E15.5 and E18.5 [(Figures 4A-H′,4I, Rb−/− vs. Rb+/− at E15.5: Sox2+; 2337+/−178 vs. 2938+/−279, (Sox2+Ki67+); 1204+/−553 vs. 900+/−358, NeuroD1+; 1624+/−146 vs. 1502+/−163, and (NeuroD1+Ki67+); 1556+/− 136 vs. 1398+/−101)]. Moreover, the number of (Sox2+AC3+) cells was similar between genotypes at E18.5, thus ruling out the presence of increased apoptosis in the stem cell population after loss of Rb (Supplementary Figure 3). Altogether, these data indicate that Rb controls proliferation of late neural precursors and early neuroblasts in the OE, e.g., iOSNs but does not seem to regulate division of stem cells or early precursors in the lineage.

**Figure 5:**
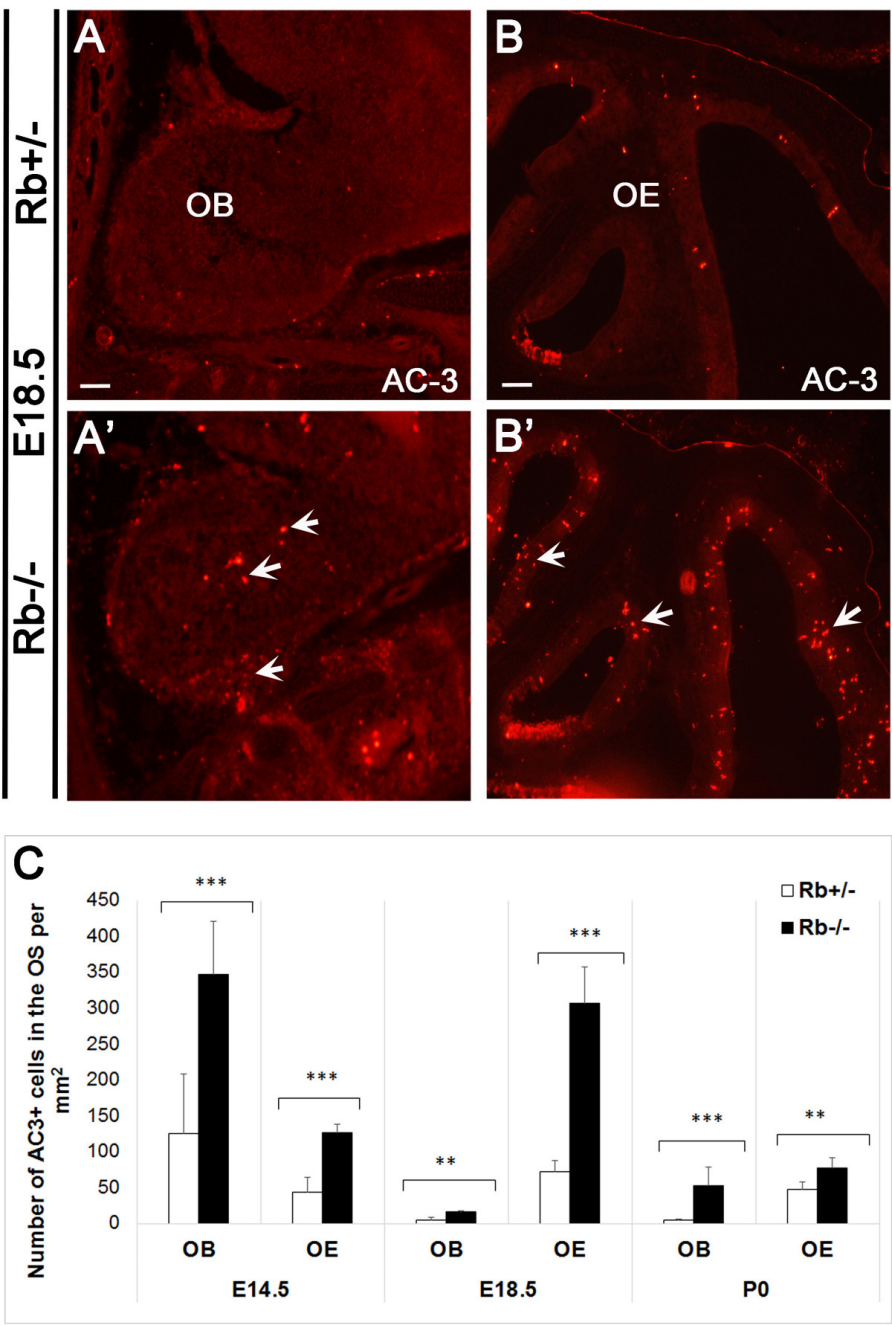
**Massive apoptosis inside the OS during late development in the absence of Rb. (A–B′)** Immunostaining against AC-3 (active-caspase 3; apoptotic marker) performed on sagittal sections and showing increased cell death in the OB **(A,A′)** and the OE **(B,B′)** in Rb−/− vs. Rb+/− embryos at E18.5 (arrows in **A**′ and **B**′). **(C)** Quantification of AC-3+ cells performed at medial levels in the OE revealed 2.94-, 4.2- and 1.63-fold increase in cell death at E14.5, E18.5, and P0, respectively, and 2.77-, 3.19- and 11.4- increase in cell death inside the OB at the same ages, respectively. Error bars represent SD of measurements from *n* = 3 per genotype and asterisks indicate a statistical significant difference between genotypes using *t*-tests, ^**^p < 0.01 and ^***^p < 0.001. Scale bars = 100 μm.

**Figure 6:**
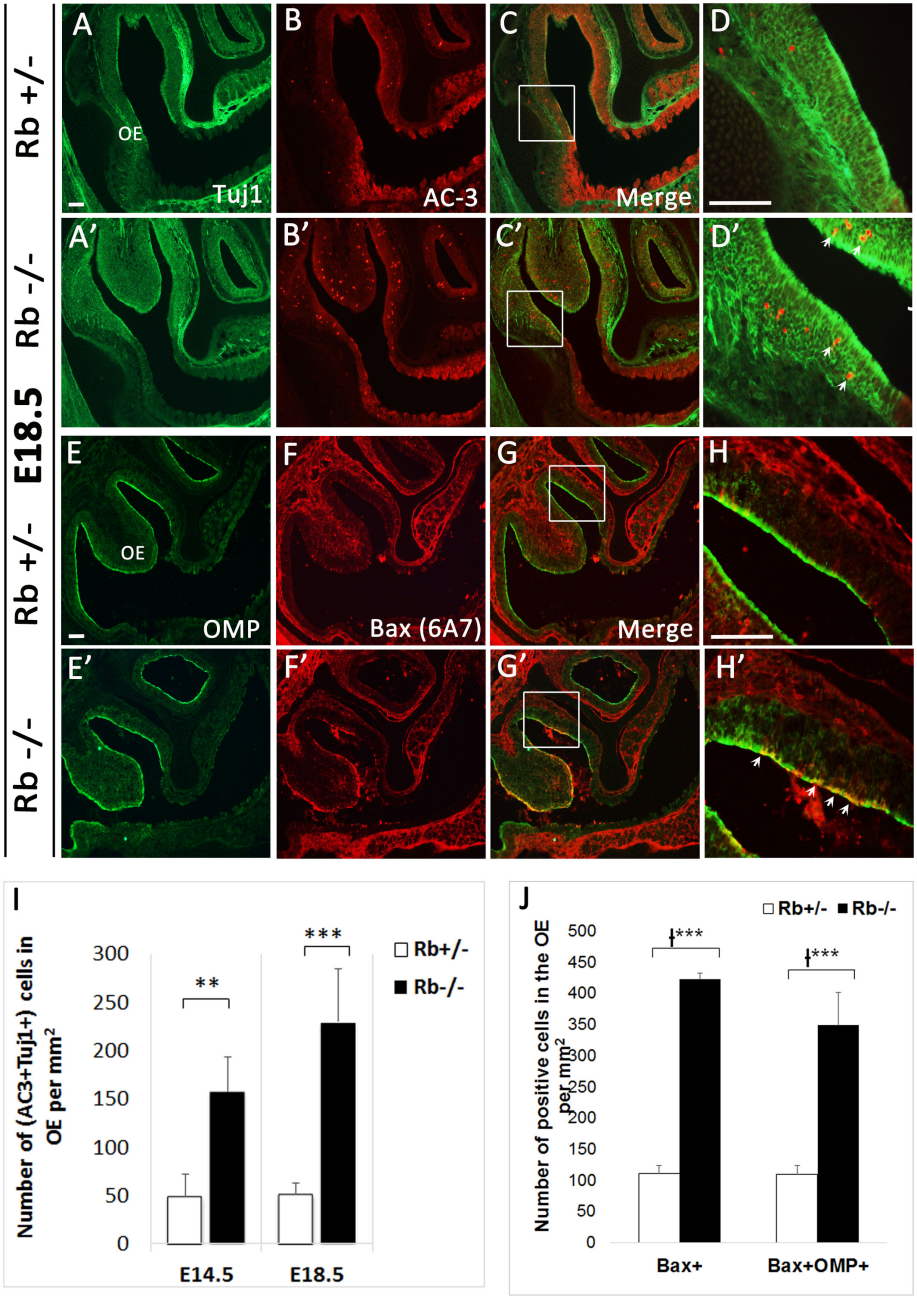
**Survival of newborn OSNs is severely compromised in the absence of Rb. (A–D′)** Double immunostaining against Tuj1 and AC-3, and **(E–H′)** OMP (Olfactory Marker Protein; marker of mOSNs) and Bax (6A7) (active form-apoptotic marker) performed on sagittal sections at E18.5. **(D–H′)** are higher magnification images of boxed regions shown in **(C–G′)**, respectively. Results showed a dramatic increase in cell death of iOSNs (arrows in **D**′) and mOSNs (arrows in **H**′) in Rb−/− vs. Rb+/− embryos (compare **D,D**′ and **H,H**′). **(I,J)** Quantification of this data showed 4.5 and 3.17-fold increase in (AC3+Tuj1+) cells **(I)** and (OMP+Bax (6A7)+) cells **(J)** at medial level inside the OE in the absence of Rb at E18.5. Error bars represent SD of measurements from *n* = 3 per genotype and asterisks indicate a statistical significant difference between genotypes using *t*-test **(I)** and 2-way ANOVA test **(J)**; ^**^p < 0.01, ^***^p < 0.001. Scale bars = 100 μm.

We next examined whether terminal differentiation and survival of OSNs is affected by the loss of Rb. Following one or more divisions, late neural precursors exit the cell cycle and differentiate into immature OSNs (iOSNs), which are located immediately apical to the GBCs in the intermediate zone (IZ) of the OE. Later on, iOSNs migrate radially to the apical surface as they upregulate the expression of the Olfactory Marker Protein (OMP) and become mature OSNs (mOSNs) with bipolar morphology (Graziadei and Graziadei, 1979; Farbman and Margolis, 1980; Calof and Chikaraishi, 1989). As a result, we found a dramatic increase by 2–4-fold in cell death in both the OE and the OB in Rb−/− vs. Rb+/− embryos between E14.5 and birth as indicated by the number of cells expressing the apoptotic marker active-caspase 3 (AC-3) (Figures 5A–C; arrows in Figures 5A′–B′, AC3+ at E14.5: Rb+/− vs. Rb−/−, 42.9+/−20.9 vs. 126.5+/−11.4, *p* = 0.00001, 2.95-fold increase in OE, and 125.16+/− 82.76 vs. 346.85+/−74.45, *p* = 0.0004, 2.77-fold increase in OB). Moreover, co-staining with (AC3/Tuj1) and (Bax (6A7)/OMP, Bax (6A7); active form-apoptotic marker) revealed significant increase in the numbers of apoptotic iOSNs between E14.5 and E18.5 in the absence of Rb (Figures 6A–D′; arrowheads in Figure 6D′, and Figure 6I; (AC+Tuj1+) at E18.5: Rb+/− vs. Rb−/−: 50.94+/−11.81 vs. 229.7+/−54.6, *p* = 0.0003, 4.5-fold increase)], and mOSNs at E18.5 [(Figures 6E–H′; arrowheads in Figure 6H′, and Figure 6J; Bax (6A7)+, Rb+/− vs. Rb−/−: 111.6+/−16.3 vs. 423.5+/−84.09, *p* = 0.0009, 3.79-fold increase, and (OMP+Bax (6A7)+): 110+/−16.8 vs. 349.7+/−66.5, *p* = 0.0002, 3.17-fold increase)]. These data indicate that survival of both iOSNs and mOSNs is severely compromised in the absence of Rb during late development.

### Deregulated expression of key signaling molecules in the OS in Rb−/− mice during late development

**Figure 7:**
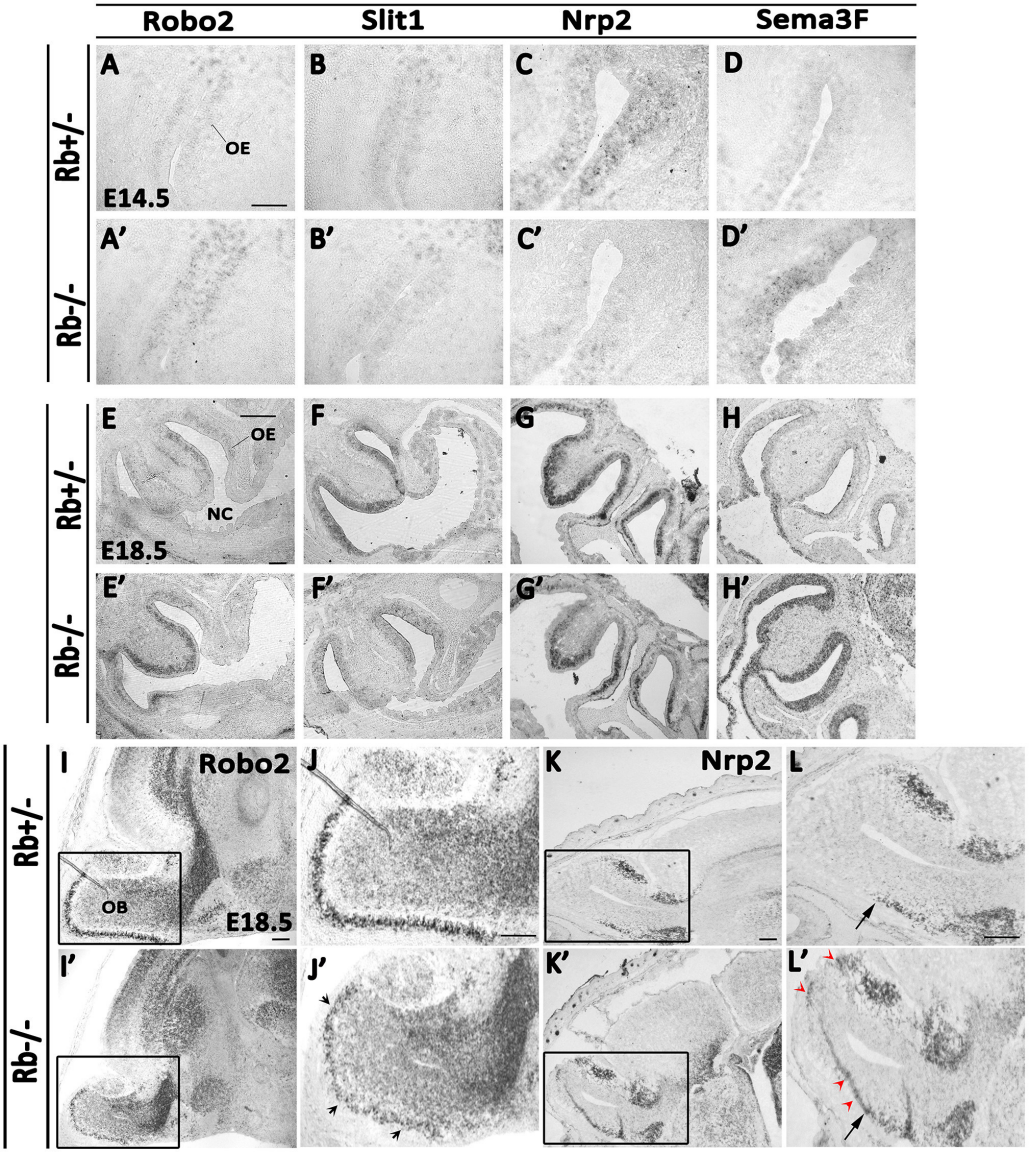
**Deregulated expression of key axonal guidance molecules inside the OS in Rb−/− mice**. The mRNA expressions of four guidance molecules, Robo2 **(A,A′,E,E′,I–J′)**, Slit1 **(B,B′,F,F′)**, Nrp2 **(C,C′,G,G′,K–L′)** and Sema3F **(D,D′,H,H′)** were assessed by *in situ* hybridization on sagittal sections at E14.5 **(A–D′)** and E18.5 **(E–L′)** in the OS. Results showed an increase in *Robo2* transcript expression and decrease in *Slit1* transcript expression in the OE in the absence of Rb. In parallel, *Sema3F* mRNA expression was increased while *Nrp2* transcript expression decreased in the OE in Rb−/− vs. Rb+/− embryos suggesting deregulated expressions of these molecules, which may explain the axonal guidance perturbations detected in the absence of Rb. Moreover, in the OB, *Robo2* mRNA expression was reduced in the GL in Rb−/− vs. Rb+/− mice (**I–J**′, black arrowheads in **J**′) whereas *Nrp2* transcript expression was extended to the dorsal OB in the absence of Rb (**K–L**′, red arrowheads in **L**′) instead of being confined to the ventral region as seen in Rb+/− controls (black arrows; **L,L**′). **(J,J′,L,L′)** are high magnification images of the regions shown in boxes in **(I,I′,K,K′)**, respectively. Scale bar = 100 μm.

The axonal guidance defects described earlier prompted us to investigate whether this phenotype could be due to impaired axonal guidance and/or axon-target interactions inside the OS. We thus examined the levels of expression of key signaling molecules between E14.5 and E18.5, the critical period when OE-OB connections are being established. We found the transcript levels of two major pairs of chemo-repellant molecules that control the topographic map of D/V OSNs projections in the OE deregulated. Hence, in the Rb−/− mice, *Robo2* mRNA expression that guides the projection of dorso-medial (D/M) OSNs axons (early-projecting axons) was increased in the OE while the mRNA expression of its repulsive ligand, *Slit1*, was significantly decreased compared with Rb+/− controls between E14.5 and E18.5 (Figures 7A–B′,E–F′). In contrast, *Robo2* transcript expression was decreased in the glomerular layer (GL) of the OB, thus suggesting the presence of a reduced synaptic connectivity between D/M OSNs axons and OB glomeruli (Figures 7I–J′; black arrowheads in Figure 7J′). In comparison, *Nrp2* mRNA expression, which guides the projection of late-projecting ventro-lateral (V/L) OSNs was decreased in Rb−/− OE while the transcript expression of its repulsive ligand, *Sema3F*, which is normally secreted by early-arriving Robo-2+ OSNs, was increased, and this was consistent with the increased *Robo2* mRNA expression in the same region (Figures 7C–D′,G–H′). Interestingly, *Nrp2* mRNA expression which is normally restricted to the ventral OB in control brains is ectopically detected in the dorsal OB in Rb−/− embryos and this is probably due to the decreased *Robo2* transcript level in this region (Figures 7K–L′; red arrowheads in Figure 7L′). These data clearly indicate that OSNs axonal guidance is severely disrupted in the OS during late embryogenesis in the absence of Rb.

## Discussion

We report here a novel requirement of Rb in the development of the primary olfactory structures and the establishment of proper axo-dendritic connections between OE-OB during development. We show that: (1) Rb controls iOSNs progenitor proliferation and their subsequent radial migration and terminal maturation, and (2) loss of Rb is associated with deregulated expression of key chemo-repellant molecules that guide mOSNs axons during the formation of D/V topographic map in the OS.

We have induced a successful deletion of Rb in the telencephalon and OS using a Foxg1-Cre driver line (Figures 1A,B). Cre expression starts around E9.5–10 in the olfactory pit and is expressed throughout the OE lineage starting in Sox2+ stem cell/precursors to iOSNs (Hebert and McConnell, 2000; Kawauchi et al., 2009). Loss of Rb, however, results in developmental defects at later stages of OS development but not earlier (Figures 1–3 and Supplementary Figures 1–3), indicating that it is either not required at earlier stages or other pocket proteins e.g., p107 and/or p130 may compensate for its loss during this period. We showed that Rb controls proliferation and radial migration of late-born OSN precursors that are Tuj1+ (iOSNs) but does not regulate the OE lineage at earlier steps such as stem cells/early precursors (Sox2+ cells) or intermediate precursors (NeuroD1+ cells) (Figures 3, 4). Hence, Rb controls the production of OSNs probably by regulating cell cycle exit in late OSNs precursors and this is consistent with its role in the dorsal cortex, the dentate gyrus and the adult subventricular zone/OB where Rb specifically regulates progenitor proliferation without affecting neural stem cells development (Ferguson et al., 2002; Ghanem et al., 2012; Naser et al., 2016; Vandenbosch et al., 2016). In contrast, while Rb is required for proper cell cycle exit and neuroblast differentiation in the dorsal cortex and the SVZ during development (Ferguson et al., 2002; Andrusiak et al., 2012; Ghanem et al., 2012), it is dispensable, at least partially, with respect to these functions in the OE (Figures 3, 4), the dentate gyrus and the adult SVZ/OB (Naser et al., 2016; Vandenbosch et al., 2016), suggesting a differential requirement for Rb in cell differentiation in distinct brain tissues. Moreover, all of the studies listed above highlighted a unanimous and systematic requirement of Rb in cell survival in the brain. Accordingly, we found that survival of both iOSNs and mOSNs is severely compromised in the Rb−/− mice compared with heterozygous controls (Figures 5, 6). While this defect may be a direct consequence of ectopic proliferation and/or abnormal migration of iOSNs precursors, loss of mOSNs late in development (and potentially other types of cells in the OS) is rather a secondary effect that is associated, at least partially, with the failure to establish proper synaptic connections between the OE-OB (see discussion below). Alternatively, our data cannot rule out that the absence of Rb in newborn OSNs could lead to sporadic cell cycle re-entry and subsequent cell death as previously observed in cortical neurons (Andrusiak et al., 2012). The control of cell differentiation and migration in different brain regions by Rb is likely to be cell-autonomous. We have previously demonstrated that the Rb/E2F pathway directly regulates *Dlx1/Dlx2* gene expression, which are two key neuronal differentiation genes in the developing brain (Ghanem et al., 2012). Moreover, using *in vivo* transplantation, Rb was shown to regulate cell migration independently of the environment in the telencephalon (Ferguson et al., 2005), a function that is specifically mediated by its interaction with E2F3 (McClellan et al., 2007) and direct regulation of a key signaling molecule, neogenin (Andrusiak et al., 2011). Interestingly, a more recent study by Wai Keung Kam et al. have revealed that neogenin and its membrane-bound ligand RGMB control progenitor cell numbers and iOSN survival in the developing OE (Kam et al., 2016). Therefore, it would be interesting to determine whether Rb controls neogenin function in the OS too.

The late developmental defects detected in the OE and OB in the absence of Rb are strongly interrelated. The incomplete penetrance of the phenotype could be due to the residual small amount of the wild type Rb protein (Figures 1A,B) and/or unknown compensatory mechanisms. Thus, many mOSNs axons fail to innervate properly distinct glomeruli within the OB during glomerulogenesis at late gestation (E15.5-P0) in Rb−/− mice (Figure 1), and this correlates with defective lamination observed in the OB (Figure 2), and causes, later on, gradual degeneration in both tissues, e.g., mitral cells and OSNs (Figures 2, 5, 6). These OE/OB defects are largely interconnected in time and mutually related in a “cause-and-effect” manner to a high degree for several reasons. In fact, OS function depends on the establishment of proper connections between OSNs axons and MCs/TCs dendrites during the critical period when the above phenotype is seen (Hayar et al., 2004; Nagayama et al., 2004; Shepherd, 2007; Gire et al., 2012). Moreover, consolidation of the OE-OB connections is only completed after birth when olfactory function is triggered [reviewed in (Mombaerts, 2006; Blanchart et al., 2011)]. Hence, failure to connect and/or re-enforce the pre-existing connection(s) may result in neuronal degeneration in both tissues as seen with MCs and GABAergic neurons (Figure 2, and Ghanem et al., 2012; Figures 2C–D′), and OSNs in Rb−/− mice (Figures 5, 6). Of note, development of the mitral layer is normal prior to the period when the coalescence of OSNs axons into protoglomerular structures is taking place [Figures 2G–G′, Ghanem et al., 2012; Figures 2E–E′; (Treloar et al., 1999; Blanchart et al., 2006)]. The axonal guidance defects and abnormal radial migration are likely associated with a cell-autonomous function of Rb (see next section). In comparison, the OB defects probably result, at least partially, from cell non-autonomous effects such as the lack of OE input to the OB during late development; however, this conclusion needs direct validation in future studies. This strongly suggests that OSNs axons may play an important role in cellular organization and synaptogenesis inside the OB particularly the MCL and GL. Previous studies have shown that the OP and/or OE contribute indeed to the induction of OB development and neurogenesis (Graziadei and Monti-Graziadei, 1992; LaMantia et al., 1993, 2000; Gong and Shipley, 1996) despite the fact that the arrival of pioneer OSNs axons from the OE occurs at E12, which postdates the genesis of mitral cells that begins at E10.5–11 (Hinds, 1968). Other studies have indicated, however, that a “primitive OB” still forms despite lack of OSN innervation, which is only required for the protrusion and lamination inside the OB (Anchan et al., 1997; Jimenez et al., 2000; López-Mascaraque and de Castro, 2002; Long et al., 2003). For example, Pax6^*sey*/*sey*^ (small eye) mutant mice lack olfactory axons but reveal features of a non-evaginated OB-like structure (López-Mascaraque et al., 1998; Jimenez et al., 2000). Yet, the failure of complete OB evagination in Pax6^*sey*/*sey*^ mutants may be attributed to a mechanism secondary to PAX6 function in the OE and the telencephalon (Anchan et al., 1997). Similarly, in *Dlx5*-deficient mice, the OB growth is initiated despite the lack of ONL, but several lamination defects are detected inside OB at E18.5 (similar to the ones reported here), again suggesting that OSN axons may still be required for OB laminar properties (Long et al., 2003). In fact, *Dlx5* transcript expression is decreased in the OB and ventral cortex in Rb−/− mice compared to Rb+/− controls during late development (unpublished observations; Ghanem N). Although this could be an indirect consequence of the reduction in *Dlx1/2* gene expression which act upstream of *Dlx5/6* and are directly regulated by Rb/E2F (Ghanem et al., 2012), it may partially contribute to the phenotype described here. The discrepancies raised above strongly imply that the interactions between OB-OE are differentially regulated in a complex spatio-temporal manner during development, and are likely associated with both cell/tissue autonomous and non-autonomous properties. Either way, the olfactory input to the telencephalon might still be essential for the complete development of the OB at least during late developmental stages.

One other key finding of this study is that, in the absence of Rb, axonal guidance, and subsequent establishment of synaptic connections between OE-OB are disrupted, and this phenotype correlates with the deregulated expression of key signaling molecules. These connections rely on reciprocal interactions between both tissues, which are mediated by specific signaling molecules and subjected to a tight spatio-temporal regulation. Hence, in Rb−/− mice, the decreased mRNA expression of the repulsive ligand *Slit1* in the ventral-lateral OE is coupled to an increased transcript expression of its receptor, *Robo2*, in the dorsal OE and decreased *Robo2* expression in the GL of the OB, suggesting that Slit1/Robo2 and possibly other chemo-attractive cue(s) which are derived from the OB and needed to guide dorso-medial OSNs axons (early-projecting neurons) are deregulated in the absence of Rb [(Figures 7, (Cho et al., 2007, 2011, 2012; Nguyen-Ba-Charvet et al., 2008; Takeuchi et al., 2010)]. Moreover, *Nrp2* mRNA expression is decreased in ventro-medial and late-projecting neurons in the OE, which likely results from the increased *Sema3F* transcript expression that is normally secreted by Robo2+ OSNs to repel Nrp2+ OSNs. Interestingly, the reverse scenario is observed in the OB where *Nrp2* mRNA expression is ectopically expanded into the dorsal OB, probably due to lower levels of *Robo2* and *Sema3F* transcripts in this region (Figures 7), implying that late-projecting OSNs are aberrantly connecting with dorsal glomeruli. Alternatively, we cannot rule out that the decreased level of expression of some guidance molecules may be attributed to the increased cell death observed in the OS. Future studies should investigate further the nature of these axonal pathfinding defects and determine whether Rb directly regulates one or more of the signaling molecules described above as previously reported for Neogenin in the dorsal cortex (Andrusiak et al., 2011). In addition, it would be interesting to determine if these defects are cell-autonomous or not by performing OE/OB explant co-cultures from Rb−/− vs. Rb+/− mice in order to check neurite growth in the presence of various chemo-attractant and chemo-repellant molecules. Finally, *Robo1* and *Slit2* are not expressed in the OE between E14.5 and E17.5, consistent with previous reports [(data not shown; Cho et al., 2007; Nguyen-Ba-Charvet et al., 2008)]. It is still unknown, however, if loss of Rb affects development of olfactory ensheathing cells which are Robo2+ glial cells found in the lamina propria outside the OE and help regulate axon guidance by associating with OSN axons and repelling *Slit* ligands that are expressed in the ventral OB (Cho et al., 2007).

This study uncovered a novel role for Rb in the control of OSNs production and survival. It also highlighted for the first time a critical role for Rb in tissue morphogenesis and reciprocal tissue interactions, particularly axonal guidance during OS development.

## Author contributions

CJ, SO, LS, and NG designed the concept of the experiments. CJ, SO, SA, NE, MN, and NG collected data, performed data analysis and interpretation. CJ and NG wrote the manuscript. NG provided financial support.

### Conflict of interest statement

The authors declare that the research was conducted in the absence of any commercial or financial relationships that could be construed as a potential conflict of interest.

## Supplementary material

Supplementary Figure 1 **Loss of Rb does not cause major developmental defects in the OE during mid-gestation. (A–B′)** Cresyl-eosin staining performed on sagittal OE sections at E12.5 in Rb+/− **(A,A′)** and Rb−/− **(B,B′)** embryos and showing similar development of the ONL between genotypes at this age. **(A′,B′)** are higher magnification images of the regions shown in black boxes in **(A,B)**, respectively. Scale Bar = 100 μm. OB; Olfactory Bulb, OE; Olfactory Epithelium, NC; Nasal Cavity, ONL; Olfactory Nerve Layer, CP; Cribriform Plate.

Supplementary Figure 2 **Proliferation and migration defects in the Rb−/− OE during late development. (A–D′)** Double immunostaining performed on E18.5 sagittal sections with Hoechst (blue), anti-Tuj1 (green), and anti-BrdU (red) in Rb+/− **(A–D)** vs. Rb−/− **(A′–D′)** embryos. **D,D**′ are higher magnification images of the regions shown in white boxes in **(B,B′)**, respectively. Loss of Rb leads to increased progenitor proliferation and abnormal radial migration in the OE. Many (Brdu+Tuj1+) are found scattered in the intermediate zone (IZ) in Rb−/− OE (arrowheads in **D**′) with randomly orientated neurites (**D**′; insets) compared with controls where they are primarily located in the apical and basal layers with parallel neurite orientation (insets in **D**).

Supplementary Figure 3 **Loss of Rb does not affect survival of stem cells and early precursors in the OE**. Graph showing no difference in the number of (Sox2+AC3+) cells in the OE between genotypes at E18.5. Counts were performed at medial levels in the OE.
