# Supplementary figures and images for: Role of Rb during Neurogenesis and Axonal Guidance in the Developing Olfactory System
Source: Front Mol Neurosci. 2016 Sep 9;9:81. doi: 10.3389/fnmol.2016.00081 (PMC5016521; doi:10.3389/fnmol.2016.00081)

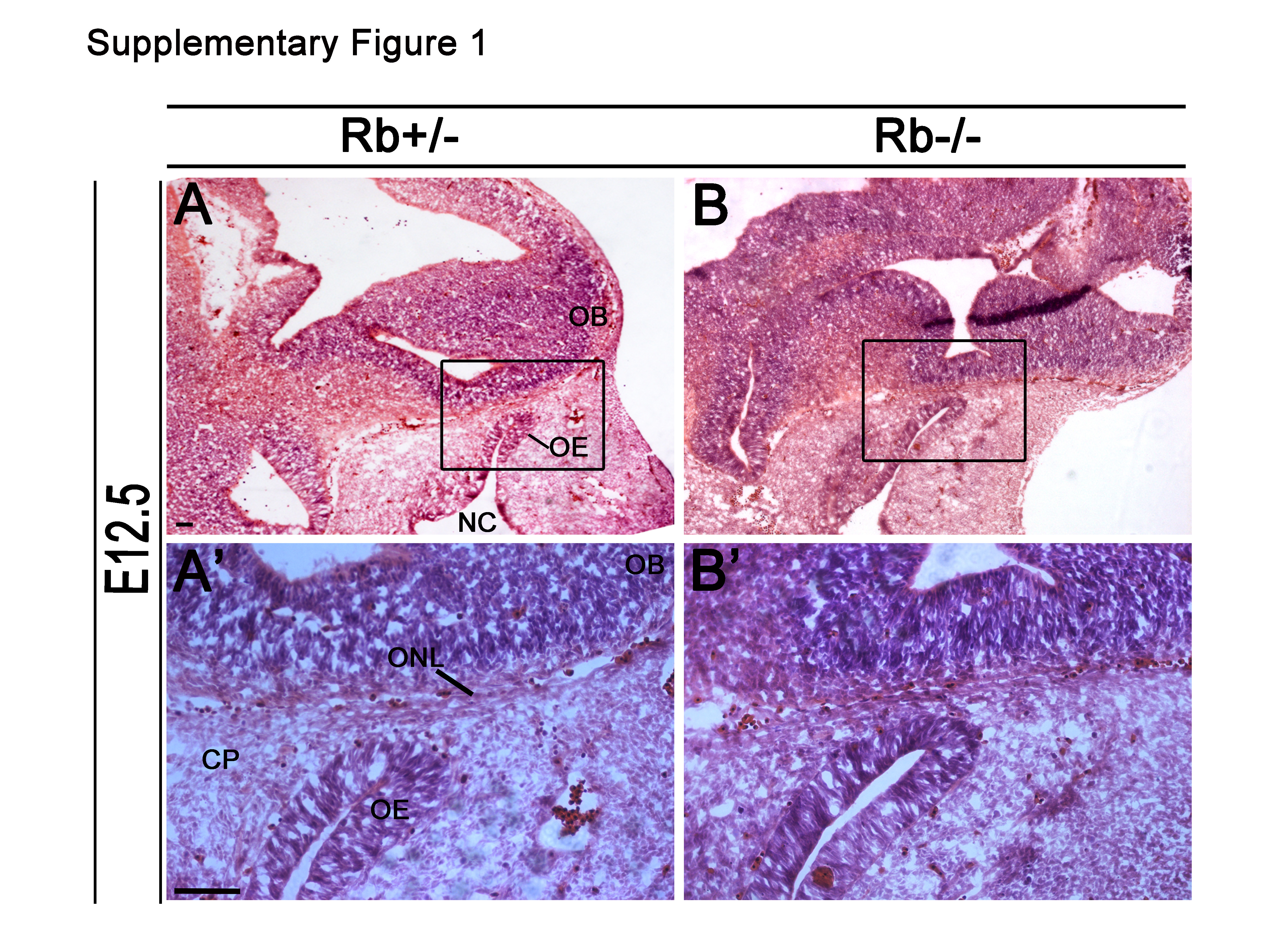

Supplement: Supplementary Figure 1 — Loss of Rb does not cause major developmental defects in the OE during mid-gestation. (A–B′) Cresyl-eosin staining performed on sagittal OE sections at E12.5 in Rb+/− (A,A′) and Rb−/− (B,B′) embryos and showing similar development of the ONL between genotypes at this age. (A′,B′) are higher magnification images of the regions shown in black boxes in (A,B), respectively. Scale Bar = 100 μm. OB; Olfactory Bulb, OE; Olfactory Epithelium, NC; Nasal Cavity, ONL; Olfactory Nerve Layer, CP; Cribriform Plate. [file Image1.JPEG]

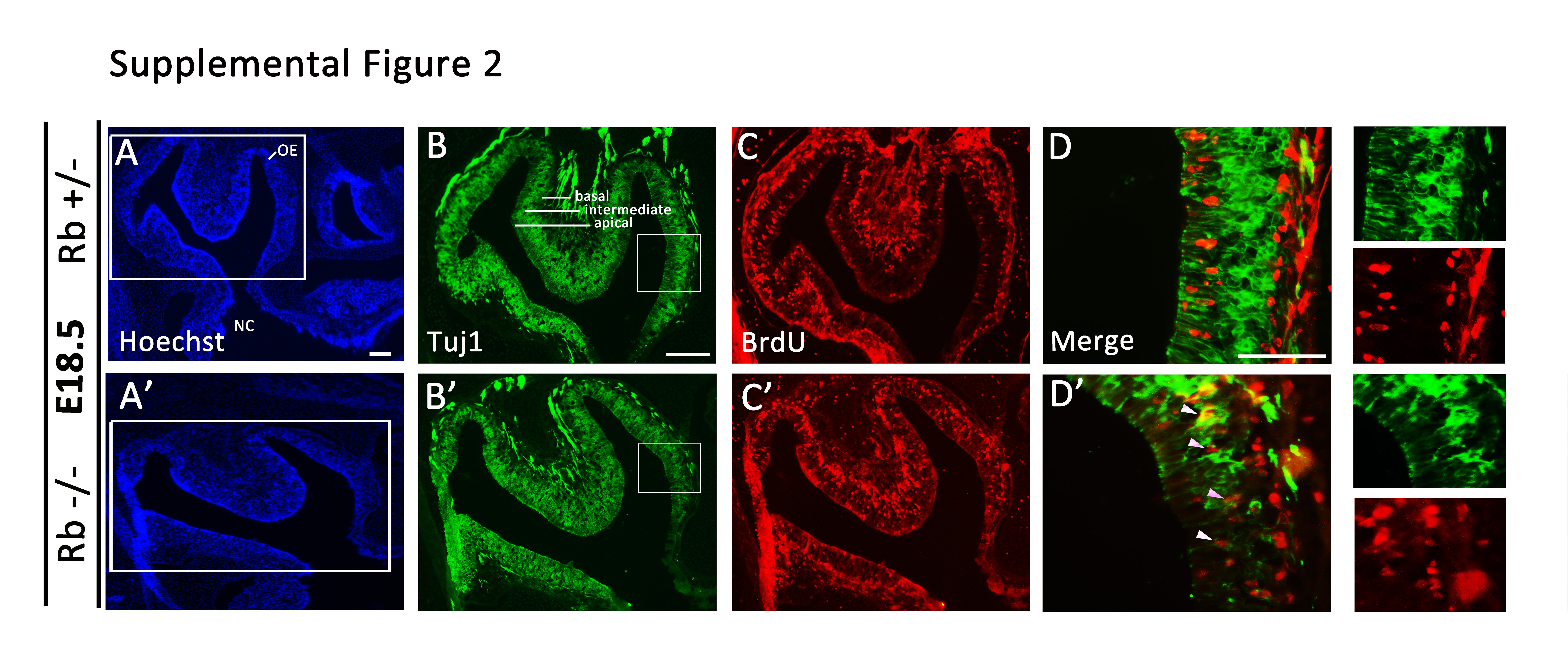

Supplement: Supplementary Figure 2 — Proliferation and migration defects in the Rb−/− OE during late development. (A–D′) Double immunostaining performed on E18.5 sagittal sections with Hoechst (blue), anti-Tuj1 (green), and anti-BrdU (red) in Rb+/− (A–D) vs. Rb−/− (A′–D′) embryos. D,D′ are higher magnification images of the regions shown in white boxes in (B,B′), respectively. Loss of Rb leads to increased progenitor proliferation and abnormal radial migration in the OE. Many (Brdu+Tuj1+) are found scattered in the intermediate zone (IZ) in Rb−/− OE (arrowheads in D′) with randomly orientated neurites (D′; insets) compared with controls where they are primarily located in the apical and basal layers with parallel neurite orientation (insets in D). [file Image2.JPEG]

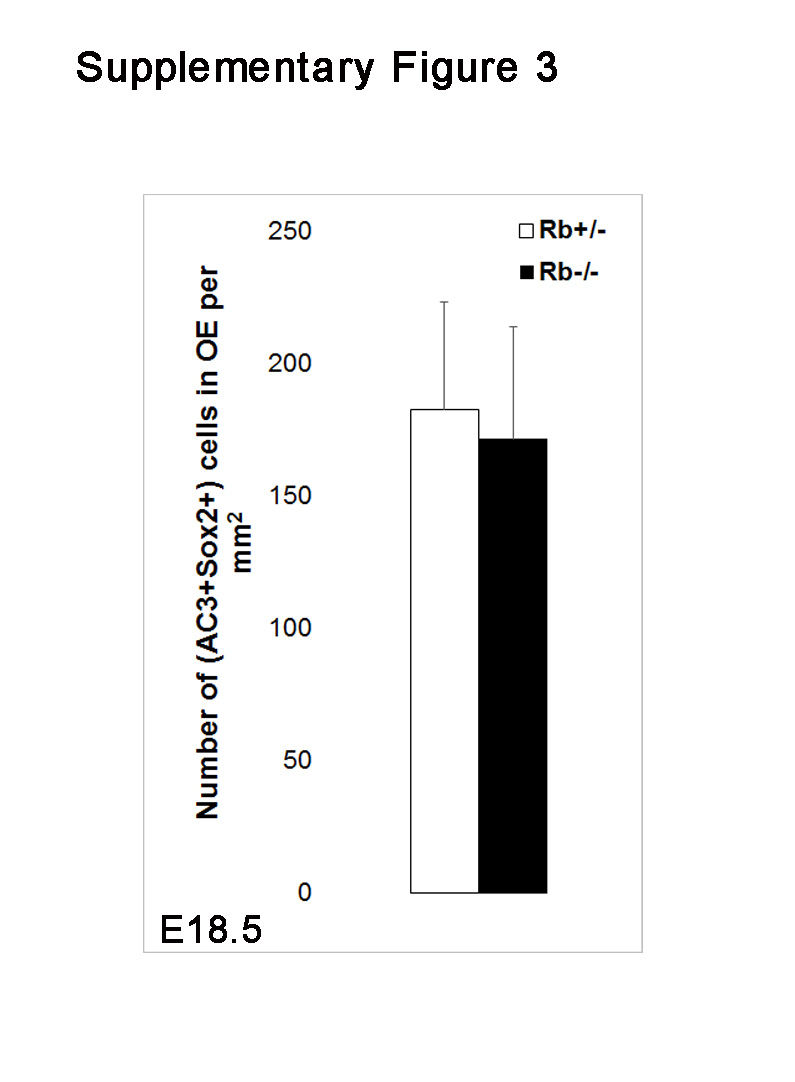

Supplement: Supplementary Figure 3 — Loss of Rb does not affect survival of stem cells and early precursors in the OE. Graph showing no difference in the number of (Sox2+AC3+) cells in the OE between genotypes at E18.5. Counts were performed at medial levels in the OE. [file Image3.JPEG]
